# Neuronal Population Activity in Spinal Motor Circuits: Greater Than the Sum of Its Parts

**DOI:** 10.3389/fncir.2017.00103

**Published:** 2017-12-19

**Authors:** Rune W. Berg

**Affiliations:** Department of Neuroscience, Faculty of Health and Medical Sciences, University of Copenhagen, Copenhagen, Denmark

**Keywords:** ensemble, spinal cord, population, CPG, motor, lognormal, fluctuation-driven, homeostasis

## Abstract

The core elements of stereotypical movements such as locomotion, scratching and breathing are generated by networks in the lower brainstem and the spinal cord. Ensemble activities in spinal motor networks had until recently been merely a black box, but with the emergence of ultra-thin Silicon multi-electrode technology it was possible to reveal the spiking activity of larger parts of the network. A series of experiments revealed unexpected features of spinal networks, such as multiple spiking regimes and lognormal firing rate distributions. The lognormality renders the widespread idea of a typical firing rate ± standard deviation an ill-suited description, and therefore these findings define a new arithmetic of motor networks. Focusing on the population activity behind motor pattern generation this review summarizes this advance and discusses its implications.

## 1. Introduction

We often assume that neurons can be categorized in homogenous and genetically well-defined groups, where each member behaves in more or less the same manner. In spinal motor research, this notion is especially appealing since genetic tools have advanced the field to the forefront of neuroscience (Machado et al., 2015; Bikoff et al., 2016; Gabitto et al., 2016; Sternfeld et al., 2017) and the cellular identity is helpful in the search for potential specialization. However, such genetic reductionism carries weaknesses as well as strengths. The sole focus on cellular subtypes has the risk of failing to see the collective properties of the network. The strategy of isolating an identified population to study its impact on behavior, for instance with genetic knock-out or optogenetics, rests on the radical assumption that the impact of this population only has a feedforward influence. Nevertheless, circuits operate by a delicate interaction between neurons of different genetic origin most likely with pervasive recurrent connectivity, where it may be fruitless to assign a role to any one member (Yuste, 2015). The intricacy of control of one population by another has recently become evident from control theory, i.e., the study of manipulation of dynamics on complex networks. The behavior of such a network is difficult to control by manipulation of selected nodes and this strategy often has counter-intuitive effects (Liu et al., 2011). A complementary investigation of the collective population dynamics is therefore recommendable in concert with genetic analysis. However, population spiking activity is a challenge not only to analyze, but also to acquire and have only rarely been done in spinal motor systems (Berg et al., 2009; Auyong et al., 2011). To achieve recordings of the population spiking activity requires multiple electrode arrays in preparations, which are mechanically stable. These challenges are part of the reason for the scarce literature and slow progress on collective properties of spinal motor networks.

### Arithmetic of population activity

**Figure 1:**
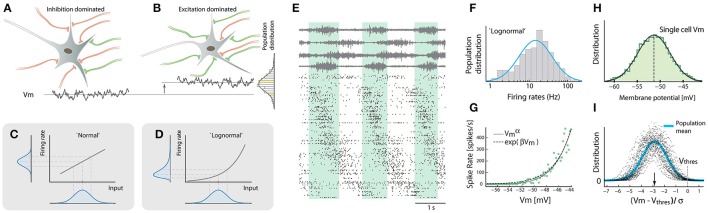
Lognormality in firing rates across neuronal population stems from a nonlinearity of the gain-curve. **(A)** Some neurons receive more inhibition (red) and others receive more excitation (green, **B**). This creates various mean *V*_*m*_ (cf. bottom traces) with a normal distribution across the population. **(C)** A normal-distributed input will be transformed to a normal-distributed output, if the gain-curve is linear. If the gain curve is exponential the output distribution will be skewed **(D)**. **(E)** Population spiking activity (~300 neurons, raster at bottom) was recorded during rhythmic motor behavior (4 motor nerves shown at top). Cycle indicated by green shaded region. Neurons sorted according to phase. **(F)** Spike count rate distribution across the population is approximately lognormal. **(G)** There is a nonlinear relationship between sub-threshold membrane potential and firing rate. **(H)** Subthreshold membrane potential strongly fluctuates with a normal distribution. **(I)** Distribution of the mean membrane potential subtracted threshold and normalized to standard deviation ((*V*_*m*_ − *V*_*thres*_)/σ) is normal across population. The typical distance of the mean to the threshold is 3σ (arrow). Adapted with permission (Roxin et al., 2011; Vestergaard and Berg, 2015; Petersen and Berg, 2016).

In a recent set of experiments, however, the mechanical stability of the turtle spinal cord (Stein, 2005) was used to investigate the neuronal population spiking activity associated with motor pattern generation within spinal circuitry (Vestergaard and Berg, 2015; Petersen and Berg, 2016). Custom-design silicon electrode arrays were used to study populations of interneurons and motoneurons in the medial-ventral portions of the lumbar region (Petersen and Berg, 2017), in order to probe the concerted activity. This yielded insight to the complexity of population spiking during motor behavior (rhythmic hindlimb scratching Petersen and Berg, 2016). Neurons were not easily sorted into discrete classes, but was rather statistically distributed as a whole, without any identifiable clustering of simple spiking behavior. Rather, the heterogeneous population revealed firing rates, which were scattered in a smooth and continuous fashion with no indication of a multimodal distribution. Further, the distribution was not the expected normal distribution with a symmetric spread around some mean, but was instead strongly skewed with a fat-tail and approximately a lognormal distribution, i.e., a normal distribution on a logarithmic x-axis (Figure 1F). Where does this lognormality come from? As it turns out, there is a simple explanation for the skewed ‘participation’ among spinal neurons. Lognormality can arise by transforming normally distributed variables with a non-linearity (Roxin et al., 2011). Here, the non-linearity is the transformation of synaptic input to a firing rate output, i.e., the gain-function, and the normally-distributed variable is the synaptic input. The net synaptic input represents the sum of excitation and inhibition, whose relative amount (or ratio) varies from neuron to neuron. Some neurons receive more excitatory than inhibitory connections (excitation-dominated) and others receive more inhibition (inhibition-dominated). Although the distribution of synaptic connections is unknown (Koulakov et al., 2009; Roxin, 2011) the resulting distribution of membrane potentials over the population is Gaussian (normal) (Figure 1). When passing such normal distribution through a non-linear response function the firing rate distribution becomes skewed. Whereas normal-distributions are typically associated with the addition of random variation (synaptic currents and potentials are roughly additive), lognormal-distributions are results of multiplicative effects (Limpert et al., 2001). The widely held belief that firing rates across the population are scattered with a mean value ± standard deviation has thus proven to be unsuitable. The network has an embedded supra-linear element. As a consequence, the addition of multiple inputs amounts to more than the linear sum of these inputs. These observations define a novel arithmetic of spinal population activity, which have previously escaped attention. As we will see below, the results also tie together population dynamics, regimes of spiking with stability and flexibility and reconciles neuronal activity in spinal cord with that found in cortex and elsewhere (Mizuseki and Buzsáki, 2013; Wohrer et al., 2013; Buzsáki and Mizuseki, 2014).

### Input is “normal” and output is “lognormal”

Neurons receive a mixture of excitatory and inhibitory synaptic connections. The precise number of connections and strength of the contacts are subject to statistical variation. Some neurons have more inhibition than excitation (Figure 1A) whereas other neurons are biased toward more excitation (Figure 1B). As a result, the mean synaptic current and membrane potential are not the same from neuron to neuron, but are rather distributed with a mean and a spread. What is the shape of this distribution? Since synaptic inputs are additive we expect a normal distribution according to the central limit theorem, both regarding net synaptic current as well as membrane potential in between spikes (Roxin et al., 2011).

Let us assume for now that the population distribution of means is normally distributed and consider two possible neuronal response functions (gain-curves). The most generic gain-curve is linear and will transform the normal distribution into a normal firing-rate distribution across the population (Figure 1C). However, if the gain-curve is non-linear the transformation will become skewed (Figure 1D). An exponential gain-curve will give an exact lognormal distribution. Other non-linearities, e.g., a power-law, will also result in skewed and “lognormal-like” distributions, although they will not be strictly lognormal. Therefore firing rate distributions are intricately linked to input distributions as well as the gain-curve.

How can a gain-curve become supra-linear? The traditional gain-curve, i.e., the frequency response to an injected current (F-I-curve) is zero below rheobase and linear above (Gerstner et al., 2014). This description is based on early intracellular measurements performed on neurons in the absence of synaptic input. Nevertheless, real input consists of rapid fluctuations and increase in synaptic conductance, which will not only change the rheobase (Grigonis et al., 2016), but also bend the gain-curve (Silver, 2010). A combination of a threshold and fluctuations in the membrane potential (or current) will thus result in an ‘expansive’ non-linearity (Hansel and van Vreeswijk, 2002; Miller and Troyer, 2002; Priebe and Ferster, 2008). The strength of such non-linearity has an inverse relationship with the degree of fluctuations (Vestergaard and Berg, 2015). Until recently nevertheless, it had not been addressed how these different elements affect the population activity in rhythm generating circuits of the spinal cord.

### Lognormality of spinal population activity

The neuronal ensemble activity of the rhythm generating circuitry of the lumbar spinal cord was therefore investigated in order to address the question (Petersen and Berg, 2016). The rhythmic hindlimb scratching of the turtle preparation was used as a model for stereotypical movement (Stein et al., 1998; Hao et al., 2014). This movement can be generated purely by the lumbar spinal cord network without the confounding factors of supraspinal input. The lack of corticospinal and bulbuspinal input also deprive the spinal circuits for certain excitatory and inhibitory input as well as neuromodulatory input such as serotonergic, adrenergic and peptidergic input. Nevertheless, several distinct behaviors can be evoked by somatic touch with no overt difference from natural behaviors (Keifer and Stein, 1983; Stein, 2005). This preparation (Petersen and Berg, 2017) offers better mechanical stability compared to in-vivo and the anoxia-resistance of this adult reptilian nervous system permits more physiologically intact activity than similar experiments in the mammalian nervous system. Thus, it was possible to monitor the spiking activity of hundreds of neurons (~300) while simultaneously recording the neuronal intracellular activity together with the motor nerve output to various hindlimb muscles (Figure 1E). The distribution of firing rates across the population closely resembled a lognormal distribution-note the log-scale (Figure 1F). Such lognormal-like distributions are also present in various other parts of the nervous system (O'Connor et al., 2010; Mizuseki and Buzsáki, 2013; Buzsáki and Mizuseki, 2014) and could represent a ubiquitous feature of neuronal networks. In order to verify the cause of this lognormality, as discussed above, the gain-curve was estimated in the subthreshold spiking (Figure 1G). The gain-curve had an expansive non-linearity similar to an exponential as well as a power-law. This non-linearity is likely due to the presence of synaptic fluctuations (Figure 1H) combined with a threshold mechanism.

Are the fluctuations, and therefore the gain-curves, different for neuron to neuron? Yes, but it was demonstrated that the non-linearity had a rather weak inverse dependence on the size of the synaptic fluctuations (Vestergaard and Berg, 2015; Petersen and Berg, 2016). This weak effect suggests that although different neurons have different levels of fluctuations in input, the non-linearity of the gain-curve is largely conserved across the population of neurons. As a consequence, the normally distributed mean membrane potential with the non-linear gain-curve together offer an explanation of the lognormality in firing rate distribution. To further verify the constancy of gain-curve across members, the membrane potentials were analyzed across a population of neurons. Since neurons have different electrotonic morphologies and thus fluctuations and thresholds, the distance of the mean membrane potential from the threshold was obtained and normalized by the size of their fluctuations (σ). This distribution ((*V*_*m*_ − *V*_*thres*_)/σ) turned out to also be normal across population (Figure 1I). Interestingly, this suggests a universal normalization of membrane potential distribution for the individual neuron to have a preferred distance to threshold of 3σ (arrow, Figure 1I). This normalization also predicts a preserved gain-curve across the population. In toto, these two elements, the normal input and the expansive non-linearity, lend support to a mechanistic explanation of the lognormal firing rate distribution (Figure 1D).

### Regimes of spiking: regular and irregular

**Figure 2:**
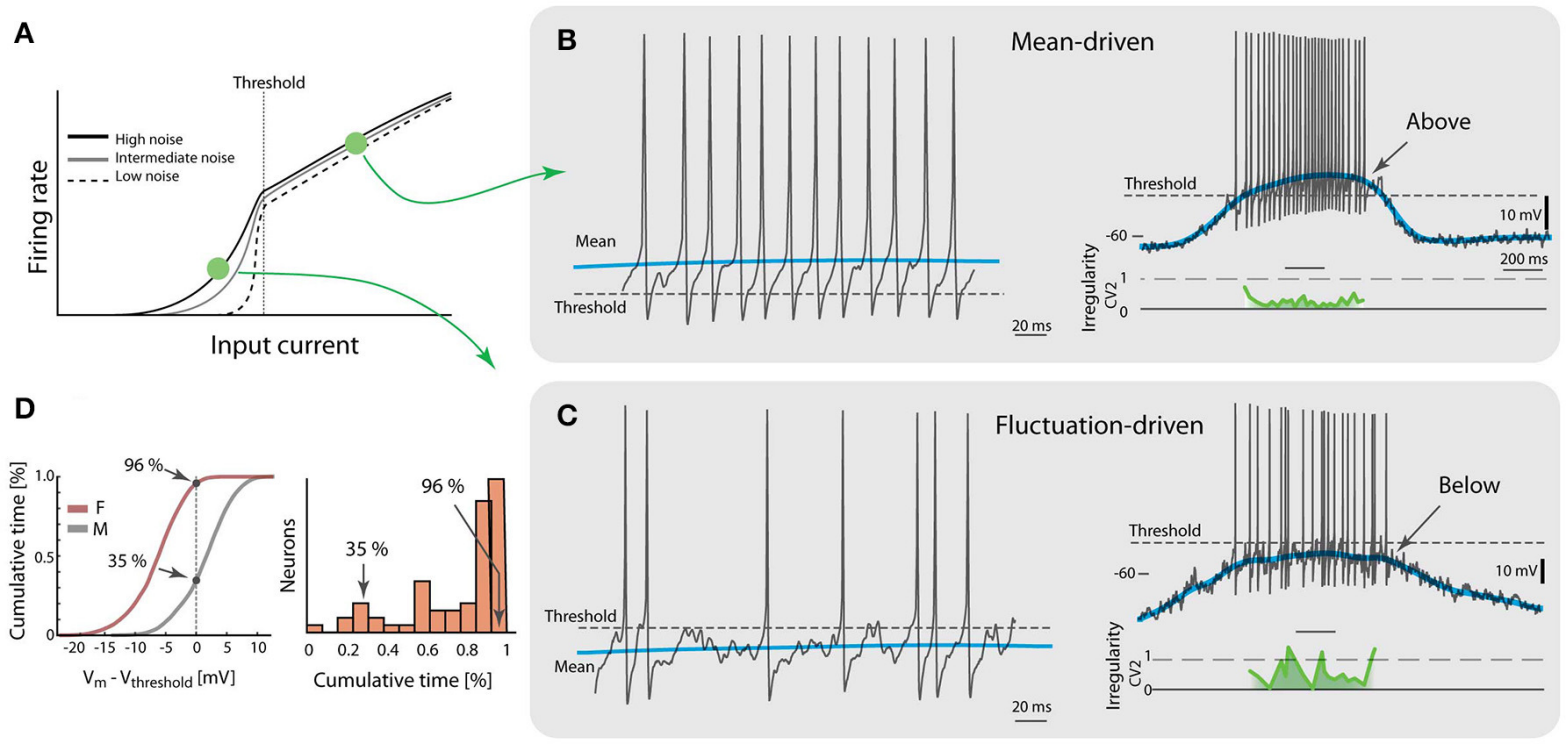
Two regimes of spiking during motor behavior. **(A)** The gain curve of a single neuron has two regimes. Fluctuations in input allow sub-threshold spiking and cause a smooth transition across threshold (vertical broken line). Larger noise causes more smooth transition. **(B)** A spinal neuron during rhythmic motor activity has mean-driven spiking, i.e., the mean membrane potential (blue line) is above threshold (broken line, left). Trace selected from a locomotion cycle (right) where most spikes have low irregularity (*CV*_2_, green trace). **(C)** Another neuron has fluctuation-driven spiking, i.e., the mean membrane potential (blue) is below threshold (broken line, left). Whole cycle indicates that majority of spikes are fluctuation-driven (right) and high irregularity (green curve). **(D)** Amount of time spent in the two regimes is quantified by the cumulative time spent below the initial threshold (left). Neuron in **(B)** has only 35% of time below threshold whereas neuron in **(C)** has 96%. Most of the neurons had a majority of time below threshold, i.e., in fluctuation-driven regime (total *n* = 68 neurons, right). Adapted with permission (Petersen and Berg, 2016).

So far, the analysis has focused on the sub-threshold spiking, where the gain-curve is non-linear and the spiking is driven by subthreshold fluctuations. How typical is this type of spiking and is there another regime of spiking? Indeed, there is another type of spiking referred to as mean-driven spiking, where the mean input current is larger than the rheobase (Renart et al., 2007; Gerstner et al., 2014). Here, the gain-curve is linear or even sub-linear (Figure 2A). The firing-rate distribution is symmetric rather than skewed (Figure 1C) although some spinal neurons have shown an increase in the gain-curve slope for much higher injected currents, i.e., the ‘secondary firing range’ due to persistent inward currents (Heckman et al., 2008; Meehan et al., 2010). The inter-spike intervals here are more affected by the after-hyperpolarizations (Matthews, 1996) and therefore the spiking is more regular and at higher firing rate (Figure 2B). This is in contrast with the fluctuation-driven spiking, which is irregular and at lower rates (Figure 2C). Some neurons spent most of the time below threshold (96% in the shown sample neuron), even without counting the inter-burst intervals, whereas other neurons spent much less time (35% in the shown sample neuron, Figure 2D). The majority of the neurons spent most of the time below threshold, i.e., in the fluctuation-driven regime (right, Figure 2D), which may be relevant for muscular control since this regime represents 90% of the force modulation in mice (Manuel and Heckman, 2011). Since the threshold has a dependence on the firing rate (Grigonis and Alaburda, 2017) the initial threshold, i.e., the threshold of the first spike in a trial, was used in this analysis. It should also be noted that the impact of intrinsic properties, such as the spike frequency adaptation, on the spiking dynamics would be difficult to assess during the these intense synaptic input. Nevertheless, intrinsic properties work on slower timescales and therefore they mainly affect spiking in a regular manner. Hence, irregularity is an indicator that can be utilized to quantify the fraction of an ensemble found in either of the regimes using spike times from extracellular recordings. Multi-electrode arrays record the extracellular potential of many neurons, and can thus more easily capture data from a larger population. Such experiments show that the neuronal population is almost equally divided between the two regimes (Petersen and Berg, 2016; see below, Figure **4**).

### Absent clustering and cellular identity

**Figure 3:**
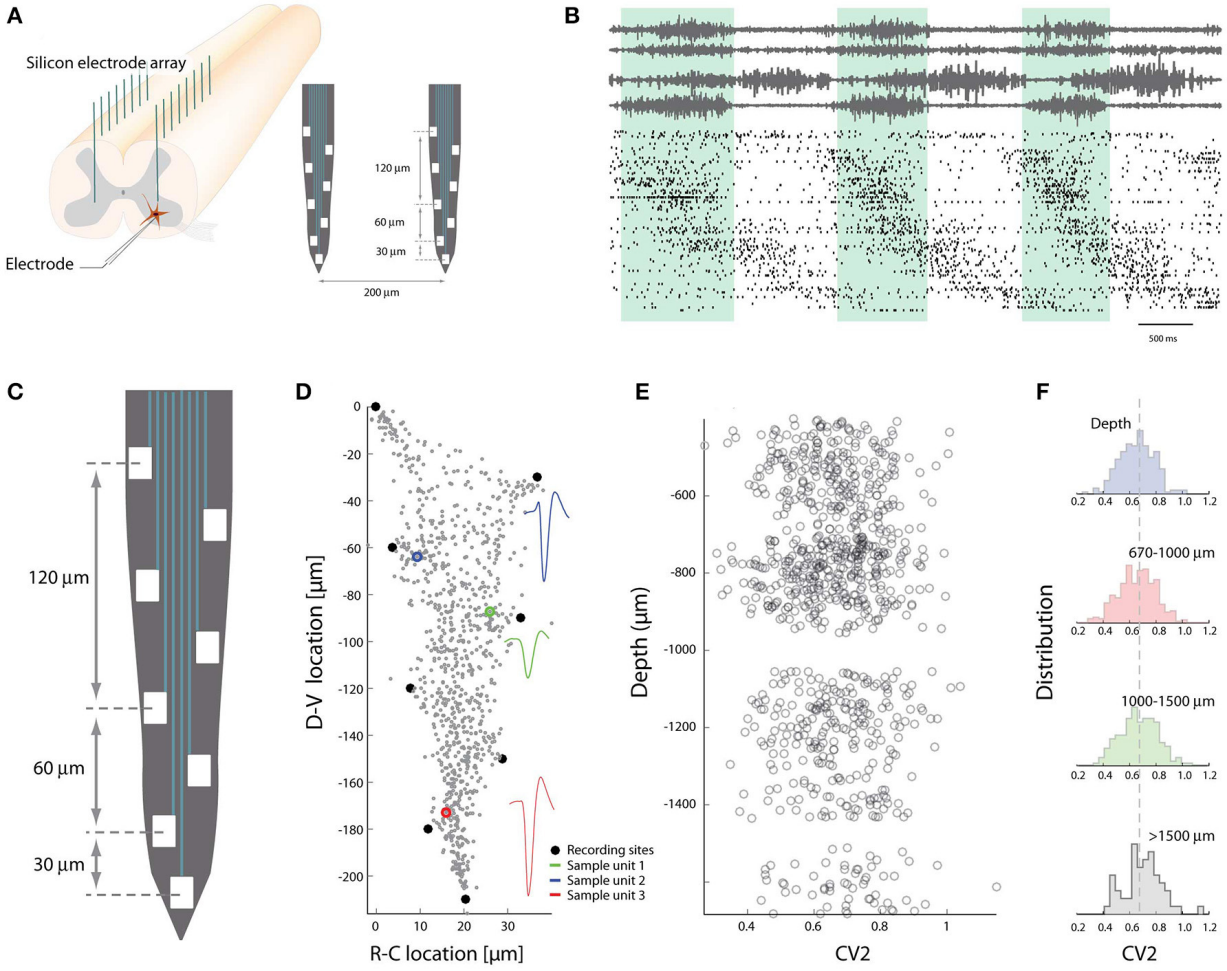
Spike patterns lack topological clustering in the dorso-ventral axis. **(A)** The multi-electrode arrays are inserted in the spinal cord gray matter along the axis of high cellular segregation (D-V direction). **(B)** Spiking pattern of sorted units (~300 neurons) concurrent with motor nerves during rhythmic movement. **(C)** Layout of electrodes on a shank. **(D)** The recording sites (black dots) are used to locate the strongest sources along the shank using trilateration. Three sample units indicated in colors, all units in gray. **(E)** The irregularity of spiking vs. depths in DV-axis. Each circle is the mean *CV*_2_ of a neuron. **(F)** The population distribution of mean spiking irregularity (*CV*_2_) as a function of depth in DV-axis. A difference between the distributions could not be detected. Adapted with permission (Petersen and Berg, 2016).

The identity of interneurons can be delineated by their genetic origin (Goulding, 2009; Hinckley et al., 2015; Kiehn, 2016). The spinal cord has the fortunate architecture that cells are developmentally segregated primarily in the dorsoventral (DV) axis of its gray matter. Therefore it is possible to probe the physiological diversity of spinal neurons by recording along the DV-axis. Custom-designed multi-channel electrodes (~256) were thus implanted in the lumbar spinal cord (Petersen and Berg, 2016) and the ensemble activity recorded (Figures 3A,B). Using trilateration and source separation combined with the shank depth it was possible to tease apart their location in the DV-axis (Figures 3C,D). The irregularity of individual members, as quantified using the *CV*_2_-measure (Holt et al., 1996), demonstrated no difference in the distribution for different location in the DV-axis (Figures 3E,F). Their distributions had simple Gaussian shapes with variance much larger than the difference in mean. This suggests that the spiking activity was equally irregular for neurons despite diversity in genetic identity. Similar observation was obtained for the firing rate distributions. These distributions were all lognormal and independent of location, indicating an absence of location-specific clustering of these simple features. Hence, there was no obvious link between genetic identity and these simple neurophysiological aspects that were characterized. This is not in conflict with another investigation that demonstrated less rhythmicity in more dorsal units (Berkowitz, 2001).

### Purpose of lognormality in spinal population activity?

**Figure 4:**
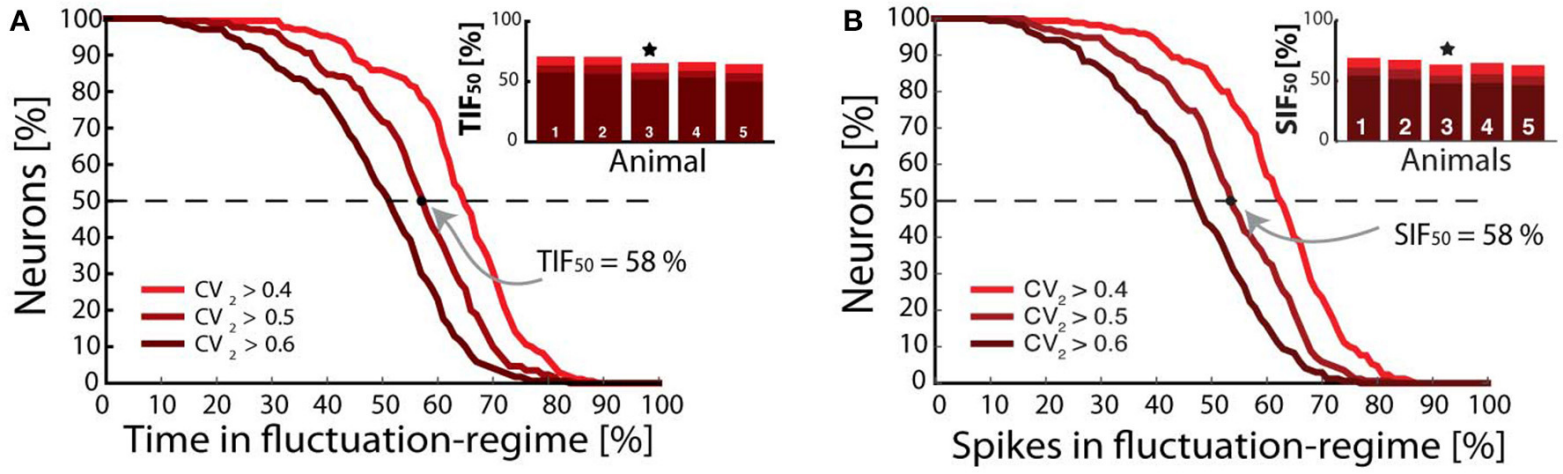
Fraction of population vs. time and spikes in fluctuation-driven regime. **(A)** Fraction of minimal time spent in the fluctuation regime (x-axis) vs. fraction of neuronal population. About half of the population spends at least 50% in with *CV*_2_ > 0.5 (broken line), i.e., Time In Fluctuation driven-regime, *TIF*_50_. Similar numbers for *TIF*_50_ was found for all animals tested (inset, *n* = 5, sample animal indicated ⋆). **(B)** Fraction of neurons vs. spikes in fluctuation-driven regime, and the similar metric Spikes in Fluctuation-driven regime *SIF*_50_. Similar numbers slightly above 50% were found for all 5 animals (inset). Adapted with permission (Petersen and Berg, 2016).

A long-standing question in theoretic neuroscience has been how neuronal networks maintain self-sustained activity while avoiding run-away excitation (Roxin et al., 2004; Kumar et al., 2008; Renart et al., 2010; Vogels et al., 2011). This is also an open question in spinal research. How do spinal circuits generate activity so reliably with high sensitivity and yet stay clear of seizures or saturation? Neural circuits most likely reach this ‘Goldilocks zone (Humphries, 2016) by stabilizing excitation via recurrent inhibition. The purpose of such an arrangement could be to ensure sensitivity to smaller input and curbing the response to strong input. The gain-curve has a sigmoidal shape, where the left part has a supra-linear summation that can amplify weak input (Rubin et al., 2015). The input can both represent sensory input or internally generated activity within the network itself. Stronger input will move up the curve to sub-linear summation, which will attenuate and stabilize activity. Such enhancement of weak input while curbing strong activity is beneficial for extending the dynamic range of the network operation. The network will always be able to increase output, although it is strenuous for stronger input. The extension of dynamical range can thus be accomplished by the sigmoidal shape of the gain curve, where each part represents the two spiking regimes. How many neurons are in the either the supra- or sub-linear part of the gain curve? The supra-linear part is exactly the fluctuation-driven regime and the sub-linear part represents the mean-driven spiking. It was therefore possible to answer this question by quantifying the fraction of the population in either of these regimes via the irregularity of spiking. Using the *CV*_2_ metric for local irregularity the time of each neuron spiking irregularly (*CV*_2_ > 0.5) the inverse cumulative distribution for the whole population measures the fraction of neurons that spike at least a certain fraction of time (x-axis) in the irregular regime (Figure 4A). The time that half of the population (broken line) spend in the fluctuation-driven regime was remarkably close to 50%. This number was conserved across animals (*TIF*_50_, inset). Similar analysis was performed for the number of fluctuation-driven spikes (spikes in fluctuation regime, *SIF*_50_) rather than time, since these might be different when the firing rate changes. Nevertheless, the analysis gave qualitatively similar results (Figure 4B). This could indicate a kind of homeostasis for network spiking activity in redundancy with cellular mechanisms, such as homeostatic plasticity (Kline et al., 2007; Pozo and Goda, 2010). Irregularity of spiking of motor neurons is not expected to be a great disadvantage since it introduces only a mild decrease in steadiness of muscle force, due to temporal summation within the muscle fibers and spatial summation of over neighboring muscle fibers, which both work as low-pass filters (Dideriksen et al., 2012).

### Future directions

An outstanding question in spinal motor research is how rhythm generation takes place in the spinal circuits (Whelan, 2010; McLean and Dougherty, 2015; Sternfeld et al., 2017). The recent findings of the ensemble activity, which I discussed in this review, do not explain rhythm generation, only certain concerted properties associated with it. Pacemaker neurons are widely believed to be responsible for rhythm generation or at least to be involved as a supporting mechanism for e.g., increasing the robustness of the oscillation (Purvis et al., 2007). Nevertheless, new findings in the respiratory field has cast doubt on pacemaker-hypothesis (Feldman et al., 2013; Feldman and Kam, 2015) although it is an on-going debate. The finding presented here that a larger portion of the population is in the fluctuation-driven regime is interesting in this context, since irregular spiking most likely originates from balanced excitation and inhibition (E/I) (Shadlen and Newsome, 1998; Petersen and Berg, 2016). Balanced E/I leaves the membrane potential close to threshold while increasing the membrane conductance sometimes several fold (Destexhe et al., 2003; Alaburda et al., 2005; Berg et al., 2007). Large increases in conductance are consequential since it can shunt the intrinsic properties (Kolind et al., 2012) and thus in principle “confiscate” the specialization of neurons (Berg and Hounsgaard, 2009). Nevertheless, conductance increase is more likely to only distort the kinetics of the pacemaker dynamics, yet still present a challenge to the pacemaker hypothesis. There are alternatives to the pacemaker-hypothesis such as network mechanisms (Grillner, 2006; Brocard et al., 2010; Li, 2011; Ramirez et al., 2011), i.e., an emerging feature on a level above single neurons (Yuste, 2015), but this hypothesis needs refinement. If the rhythm is generated by the population rather than individual pacemaker cells, ensemble recordings—as presented in this review—are crucial for understanding rhythm generation (Shenoy et al., 2013; Bruno et al., 2015, 2017). Therefore, the fact that experiments on population spiking activity are so rare suggests a research area worth while of pursuing. Optical imaging of the calcium activity of neuronal populations in the spinal cord (Ritter et al., 2001; Wilson et al., 2007; Kwan et al., 2010; Johannssen and Helmchen, 2013; Renninger and Orger, 2013; Weinger et al., 2015) are an important tool to include, especially with DNA-encoded calcium indicators, which can specifically label subpopulations of neurons (Muto et al., 2011; Machado et al., 2015). Obstacles in optical imaging are the lower temporal resolution as well as the relativity in signal (Δ*F*/*F*), which each prevents an analysis, similar to the one presented here, to be performed. Functional connectivity in association with multi-electrode recordings can assist in mapping the network in spinal cord behind motor pattern generation, which is largely unknown. Although multi-electrode recordings in mammalian spinal cord remain a challenge (Berg et al., 2009; Auyong et al., 2011) a natural next step is to verify these findings in the mammalian spinal cord.

## Author contributions

The author confirms being the sole contributor of this work and approved it for publication.

### Conflict of interest statement

The author declares that the research was conducted in the absence of any commercial or financial relationships that could be construed as a potential conflict of interest.
